# Physiological Basis of Labor

**Published:** 1893-06

**Authors:** 


### PHYSIOLOGICAL BASIS OF LABOR.

That there are practical difficulties in the adoption of an eight hours' day for all workers none can deny; whether workers are likely to make a proper use of spare time, and whether an eight hours' day should be compulsory or optional, are matters on which there is great difference of opinion. Few will question that grinding toil from morning till night is most undesirable, and that a reasonable time for recreation and change of scene is necessary for all workers, not least for those whose employment calls into play none of the higher and intellectual sides of the mind, and for those whose work, like that of miners, is of an uninviting and laborious nature.

It is impossible, however to argue such questions from the mere lofty standpoint of theory. Labor problems cannot always be put to the touch of experiment; but in this particular case the experiment has been tried, and its success will do more to convince objectors than any amount of theoretical quibbling. Certain engineering firms in the north of England having determined to give the eight hours' day a trial, took the precaution to agree with the men to reduce their wages by such an amount as would cover half the anticipated loss, the masters bearing the other half. After a few months it was found that there was no loss whatever; the output was as large as before.

Such a result, and it is by no means unique, is not only intensely interesting to the parties immediately concerned, but also opens up a field of physiological research which is almost untouched. It is not often that politics and physiology come into such close relationship. It amounts to this, that when the men worked for nine or ten hours, one or two hours were to all practical intents and purposes wasted.

We do not mean deliberately wasted, but that the natural processes of fatigue operated in such a manner as to lead to a wasteful expenditure of energy. In a shorter day the workmen work with a will, to put it popularly; that is to say, the hours are not sufficiently long to admit of the onset of a time when the voluntary control over the muscles is necessarily lost to a great extent.

The question of fatigue is very nearly related to that of the muscular sense, and few physiological questions have been more keenly discussed. Muscular fatigue is not a purely local muscular condition; the nervous centres are also at fault. Mosso, of Turin, has gone so far as to suppose that some toxic substance is produced during muscular activity which, passing into the blood and reaching the brain, impairs its activity and the will power associated therewith. He has also, by means of an instrument which he has devised aud named the ergograph, shown graphically that the fall in the amount of voluntary contractions is not necessarily a steady one, but may exhibit rises in the course of the downfall.

Returning now to the operatives and their eight hours' day, the question arises what is the curve that would represent graphically the amount of work done as the day progresses ? Here our information is necessarily scanty ; observations are at present few in number, and the investigation on a mass of mill hands is necessarily difficult. In one case, of which we have received private information, the rough and ready method was adopted of directing the foreman to estimate the output per hour.

It was found that the least productive period was the time before breakfast ; after this meal the efficiency of the work gradually increased, and reached a maximum about 11 in the morning. From this time onward there was a steady fall, uninfluenced apparently by the subsequent meals, until the end of the day.

This is perhaps what one would expect ; working on an empty stomach is not working under the most advantageous circumstances, in spite of the long period of rest by which it is preceded. But every pedestrian knows that his most successful efforts are not made at the start, but when he gets warmed to his work ; and every student who has taken muscle curves is aware of what is called the "beneficial effect of contraction," which precedes the occurrence of fatigue.

Dr. Warren P. Lombard, of Worcester, Mass., has made, by Mosso's method, some experiments on himself, in which he has confirmed many of Mosso's results, as well as discovered some new facts. Experiments on a single individual will have to be controlled and, doubtless, corrected by observations on others, but as these are the most recent exact experiments performed in this field of scientific research, it may not be uninstructive to quote some of his more important results.

He finds that the causes of fatigue in the muscles made to contract voluntarily are to be sought chiefly in the central nervous system ; this is followed by rapid recuperative power. Those influences which lessen the power to do voluntary work are hunger, a fall of barometric pressure, a high temperature, a moist atmosphere, and the use of tobacco.

Those which increase the ability to do voluntary muscular work are exercise, rest, and especially sleep, food, and rise in atmospheric pressure, and the use of alcohol. The experiments with alcohol taken in small quantities were, however, few in number, and did not last sufficiently long to show more than the primary stimulating effect of the drug.

As we said at the outset, observations such as these open up a wide and interesting field, and if followed up on a larger scale by more accurate observations on the working power of larger masses of operatives, we doubt not that social reformers may discover that there is a physiological basis for the shortening of working hours.—*Exchange.*

